# Cancer incidence among children and young adults who have undergone x-ray guided cardiac catheterization procedures

**DOI:** 10.1007/s10654-018-0357-0

**Published:** 2018-01-18

**Authors:** Richard W. Harbron, Claire-Louise Chapple, John J. O’Sullivan, Choonsik Lee, Kieran McHugh, Manuel Higueras, Mark S. Pearce

**Affiliations:** 1 Institute of Health and Society, Sir James Spence Institute, Royal Victoria Infirmary, Newcastle University, Newcastle upon Tyne, NE1 4LP UK; 2 NIHR Health Protection Research Unit in Chemical and Radiation Threats and Hazards, Newcastle University, Newcastle upon Tyne, NE2 4AA UK; 3 Regional Medical Physics Department, Freeman Hospital, Newcastle-upon-Tyne Hospitals NHS Trust, Newcastle upon Tyne, NE7 7DN UK; 4 Paediatric Cardiology, Freeman Hospital, Newcastle upon Tyne Hospitals NHS Foundation Trust, Newcastle upon Tyne, NE7 7DN UK; 5 Division of Cancer Epidemiology and Genetics, National Cancer Institute, National Institutes of Health, Bethesda, MD USA; 6 Radiology Department, Great Ormond Street Hospital for Children NHS Trust, London, UK; 7 Basque Center for Applied Mathematics, Alameda de Mazarredo, 14, 48009 Bilbao, Basque Country Spain

**Keywords:** Cardiac, Radiation exposure, Dose, Transplant, Cancer

## Abstract

Children and young adults with heart disease appear to be at increased risk of developing cancer, although the reasons for this are unclear. A cohort of 11,270 individuals, who underwent cardiac catheterizations while aged ≤ 22 years in the UK, was established from hospital records. Radiation doses from cardiac catheterizations and CT scans were estimated. The cohort was matched with the NHS Central Register and NHS Transplant Registry to determine cancer incidence and transplantation status. Standardized incidence ratios (SIR) with associated confidence intervals (CI) were calculated. The excess relative risk (ERR) of lymphohaematopoietic  neoplasia was also calculated using Poisson regression. The SIR was raised for all malignancies (2.32, 95% CI 1.65, 3.17), lymphoma (8.34, 95% CI 5.22, 12.61) and leukaemia (2.11, 95% CI 0.82, 4.42). After censoring transplant recipients, post-transplant, the SIR was reduced to 0.90 (95% CI 0.49, 1.49) for all malignancies. All lymphomas developed post-transplant. The SIR for all malignancies developing 5 years from the first cardiac catheterization (2 years for leukaemia/lymphoma) remained raised (3.01, 95% CI 2.09, 4.19) but was again reduced after censoring transplant recipients (0.98, 95% CI 0.48, 1.77). The ERR per mGy bone marrow dose for lymphohaematopoietic neoplasia was reduced from 0.541 (95% CI 0.104, 1.807) to 0.018 (95% CI − 0.002, 0.096) where transplantation status was accounted for as a time-dependent background risk factor. In conclusion, transplantation appears to be a large contributor to elevated cancer rates in this patient group. This is likely to be mainly due to associated immunosuppression, however, radiation exposure may also be a contributing factor.

## Introduction

Information on cancer rates among children and young adults (< 22 years) with congenital or acquired heart conditions is limited. A small number of studies have been published [1–7], suggesting relatively high cancer incidence and mortality, compared to the general population. Potential explanations include shared genetic or environmental factors, immunosuppression, lifestyle, and radiation exposure [3, 5]. Children with heart disease are subjected to a number of forms of medical x-ray examinations, including computed tomography (CT), cardiac catheterizations and general radiography [8–11]. Although radiographs comprise the majority of procedures, the majority of the cumulative radiation dose in this patient group (around 80%) comes from CT and catheterizations [8, 12]. Diagnostic medical radiation exposure has increased substantially in recent decades among this patient group [9]. The long-term impact of this exposure is difficult to determine, however. In this study, we established a cohort of 11,270 patients who had undergone cardiac catheterizations before 22 years of age. We then conducted two analyses; (1) an overall assessment of the incidence of all malignant tumours in the cohort, and (2) an assessment of the potential contribution of radiation exposure and organ transplantation on cancer rates.

## Materials and methods

The study received a favourable ethical opinion from the National Research Ethics Service Committee North East—Newcastle and North Tyneside 2 Ethics Committee, along with approval from the Confidentiality Advisory Group to use patient identifiable data.

**Fig. 1:**
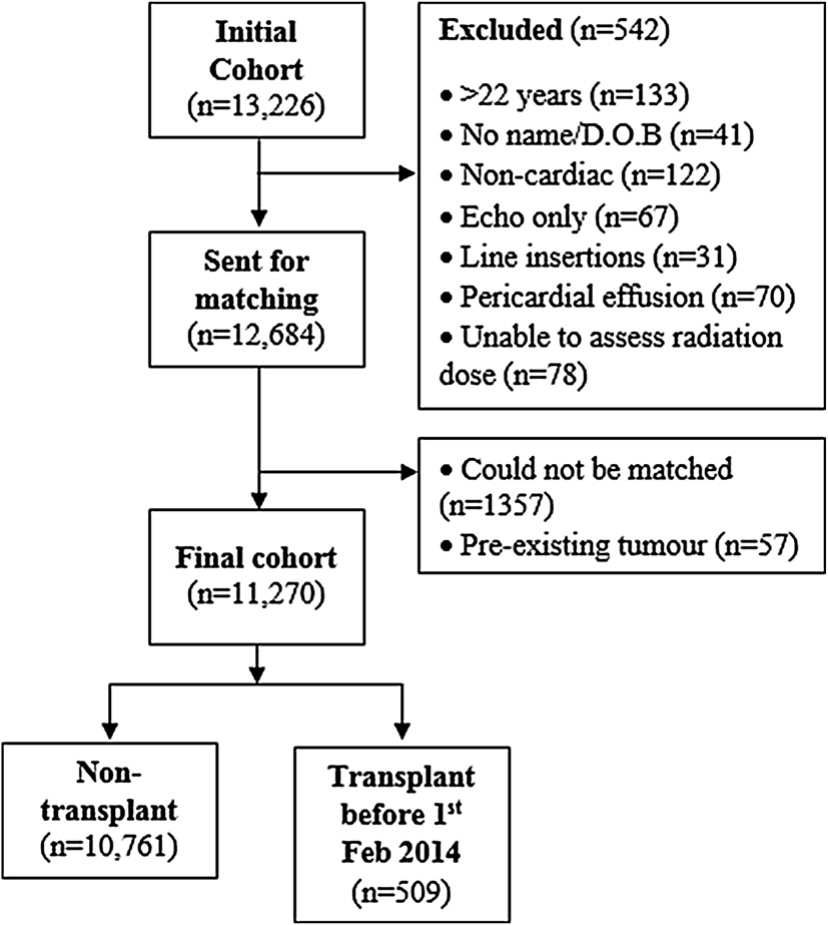
Flow diagram for cohort

A retrospective cohort was created from hospital records of examinations carried out in catheterization laboratories at five participating English hospitals. Patients were eligible for inclusion if they had undergone at least one cardiac catheterization procedure while aged under 22 years. An initial cohort of 13,226 individuals was established. Patients lacking full name or date of birth (n = 41), those who were over 22 years old at the time of the first recorded procedure (n = 133) along with patients who had undergone only trans-oesophageal echocardiography (n = 67), non-cardiac fluoroscopy procedures (n = 122), Hickman or other line insertions without any other cardiac catheterization procedures (n = 31) were excluded, as were those for whom radiation doses could not be estimated (n = 78). Patients who had undergone isolated pericardial effusion drainage (n = 70) were also excluded. This left a cohort of 12,684 individuals which was matched with the National Health Service Central Register (NHSCR) to determine who had been diagnosed with a neoplasm (reported as ICD-0 codes), the date of diagnosis, and date and cause of death, where applicable. Fifty seven patients were diagnosed with a neoplasm before the date of their first recorded cardiac catheterization. These patients were excluded from the analysis to reduce the impact of reverse causality (i.e. malignancy or associated treatment causing heart disease). 1357 cohort members could not be matched by NHSCR. This left 11,270 patients who were included in the analysis (Fig. 1). Details of this final cohort are shown in Table 1.

**Table 1:** Details of cohort

Characteristic	Whole cohort	Transplant recipients	Cases^a^
Sex			
Male (% of whole cohort)	5612 (50%)	247 (49%)	36 (49%)
Female	5118 (45%)	242 (48%)	34 (47%)
Unknown	540 (5%)	20 (4%)	3 (4%)
Total	11,270	509	73
Patient age/year of birth			
Median age at 01/02/2014 [10th, 90th percentiles]	13.5 y [3.0, 25.1]	18.9 y [8.0, 28.7]	24.0 y [8.5, 33.7]
Born < 1980 [% of total]	217 [2%]	27 [5%]	9 [12%]
Born 1980–1989	1346 [12%]	135 [27%]	35 [48%]
Born 1990–1999	4156 [37%]	209 [42%]	17 [23%]
Born 2000–2009	4486 [40%]	118 [24%]	12 [16%]
Born > 2010	1065 [9%]	11 [2%]	0
Cardiac catheterizations			
Median age at first recorded procedure [10th, 90th percentiles]	3.2 y [0.1, 15.5]	9.0 y [1.1, 16.8]	11.6 y [0.9, 17.9]
Mean number of procedures [median, 10th, 90th percentiles]	1.5 [1, 1, 3]	4.5 [1, 4, 10]	3.4 [1, 2, 8]
1 procedure [% of total]	8489 [75%]	124 [25%]	36 [49%]
2 procedures	1555 [14%]	67 [13%]	9 [12%]
3 procedures	580 [5%]	57 [11%]	7 [10%]
4 procedures	229 [2%]	44 [9%]	2 [3%]
5 procedures	150 [1%]	53 [11%]	5 [7%]
> 5 procedures	267 [2%]	155 [31%]	14 [19%]
Computed tomography			
Median age at first recorded scan [10th, 90th percentiles]	5.1 y [0.1, 17.8]	8.7 y [1.2, 17.7]	9.9 y [2.4, 20.6]
Mean number of procedures [median, 10th, 90th percentiles]	0.4 [0, 0, 1]	2.7 [1, 0, 8.2]	4.9 [1, 0, 14]
No scans [% of total]	9666 [86%]	238 [47%]	35 [48%]
1 scan	760 [7%]	74 [15%]	7 [10%]
2 scans	352 [3%]	47 [9%]	2 [3%]
3 scans	187 [2%]	32 [6%]	3 [4%]
4 scans	84 [1%]	24 [5%]	2 [3%]
5 scans	60 [1%]	19 [4%]	4 [5%]
> 5 scans	161 [1%]	75 [15%]	20 [27%]

*SD* standard deviation, *y* years

^a^Malignant, borderline malignant and benign tumours

Standardized incidence ratios (SIR) of malignancies, with associated confidence intervals (CI), were calculated as the ratio of observed to expected cases. Expected incidence, from the date of the first cardiac catheterization, up to the 1st of February 2014 was estimated from data published by Cancer Research UK [13], representing sex- and age-specific UK-wide rates. Observed/expected central nervous system tumours included benign or borderline malignant tumours (ICD codes D32-D33, D35.2-D35.4, D42-D43, and D44.3-D44.5). However, these non-malignant/borderline diseases, along with non-melanoma skin cancer (NMSC), were not included in the observed/expected figures for the analysis of all malignancies combined.

As organ transplantation, with associated immunosuppressant drug use, is also a known risk factor in cancer development [14], we matched our cohort with the NHS Transplant Registry, to determine which individuals had received a transplanted organ, the organ involved, and the date of transplant. We also examined notes fields in procedure logbooks (available for 55% of cohort members) and NHSCR death records for further information on transplantation.

To assess the possible role of radiation exposure on cancer incidence, expected cases were also calculated from 5 years following the first recorded cardiac catheterization (2 years for leukaemia and lymphoma) to 1st February 2014. These represent the apparent minimum latency periods for radiation induced tumours, based on previous epidemiological studies [15].

For cardiac catheterizations, active bone marrow (ABM) doses were estimated for 79% of examinations from dose indicators recorded at the time of each procedure (kerma area product in 71% and screening time for 8%), using a dosimetry system based on Monte Carlo computer simulations (PCXMC V2.0, STUK, Helsinki, Finland). This incorporated information on typical values of x-ray energy, beam projection angle and field size extracted from a review of clinical images and structured dose reports. For the remaining 21% of examinations for which no dose indicator was recorded, estimated doses were calculated based on the median doses for each procedure type at the same hospital, using the same equipment, in which dose indicators were recorded. Information on CT scans was obtained from a cohort of children and young adults scanned in Great Britain between 1983 and 2013 [16]. CT doses were estimated based on the methodology of Kim et al. [17], taking into account the body part scanned, patient age and year of scan (technological developments have led to a reduction in doses over time).

The excess relative risk (ERR) of lymphohaematopoietic neoplasia (leukaemia and lymphoma, including borderline malignancies) in relation to cumulative ABM dose was calculated using Poisson regression models, fitted by maximum likelihood estimation, using the maxLik function in R (R Foundation for Statistical Computing, Vienna, Austria). The expected number of cases in stratum *i* was assumed as:

$$PY_{i} \exp \left( {\alpha_{0} + \alpha_{1} 1_{{0 \le a_{i} < 5}} + \alpha_{2} 1_{{5 \le a_{i} < 10}} + \alpha_{3} 1_{{10 \le a_{i} < 15}} + \alpha_{4} 1_{{15 \le a_{i} < 20}} + \alpha_{5} 1_{{20 \le a_{i} < 25}} + \alpha_{6} 1_{{a_{i} \ge 25}} + \alpha_{7} T_{i} } \right) \cdot \left( {1 + \beta_{1} \cdot D_{i} + \beta_{2} \cdot T_{i} \cdot D_{i} } \right);$$

where $PY_{i}$, *a*_*i*_ and *D*_*i*_ are the number of person-years of follow-up, the average attained age and the accumulated dose (in mGy) in stratum *i*, respectively. The covariate *T*_*i*_ represents transplantation status. This regression function is the product of the person-years as the offset, the exponential term which represents the baseline rate and the relative risk associated with the absorbed dose. The parameter *α*_7_ represents the risk factor for transplant status, while *β*_1_ represents the ERR per mGy and *β*_2_ represents the ERR transplantation variation effect. Person-years were calculated using the DATAB module of Epicure (HiroSoft International Corporation, Seattle, USA). Both exclusion and cumulative ABM dose lag periods were of 2 years.

## Results

Across the whole cohort, 40 patients developed a malignant neoplasm following their first recorded cardiac catheterization, after 92,629 years of follow-up (mean = 8.4 years). One patient developed two distinct diseases on different occasions, giving a total of 41 malignancies, of which 30 (73%) were leukaemia (n = 7) or lymphoma (n = 23) (Table 2). There were no malignancies of the breasts, lungs, stomach or oesophagus. The SIR was raised, compared to UK-wide background rates (Table 3), for all malignancies combined (2.32, 95% CI 1.65, 3.17) and lymphoma (8.34, 95% CI 5.22, 12.61). The SIR was also raised for leukaemia, though with a wide confidence interval (2.11, 95% CI 0.82, 4.42).

**Table 2:** Details of neoplasia (malignant or otherwise) diagnosed among cohort members

Neoplasm type:	Whole cohort			Post-transplant	
	Total	Malignant	Borderline or benign	Malignant	Borderline or benign
lymphohaematopoietic	44	30	14	23	10
Hodgkin’s lymphoma	4	4	0	4	0
Non-Hodgkin’s lymphoma	19	19	0	19	0
Lymphoproliferative disorder	9	0	9	0	9
Acute lymphoblastic leukaemia	3	3	0	0	0
Acute myeloid leukaemia	3	3	0	0	0
Other leukaemia	1	1	0	0	0
Other haematological neoplasia	5	0	5^a^	0	1
Sarcoma	3	2	1^b^	0	0
Central nervous system	4	2	2	1	0
Carcinoma/carcinoma in situ	16	4	12	2	3
Cervix/exocervix	10	1	9	0	0
Testes	2	2	0	0	0
Melanoma	1	1	0	0	0
Other	4	0	4	0	0
Total	74^c^	41	33	26	13

^a^Includes polycythaemia vera

^b^Uncertain diagnosis (dermatofibroma/fibrosarcoma)

^c^One patient developed two diseases

**Table 3:** Standardized incidence ratio (SIR) for malignancies developing between the date of each patient’s first recorded cardiac catheterization and 1st February 2014

Disease	Observed	Expected	SIR [IQR]
*All patients*			
All malignancies	41	17.66	2.32 [1.65, 3.17]
Leukaemia	7	3.31	2.11 [0.82, 4.42]
Lymphoma	23	2.76	8.34 [5.22, 12.61]
Hodgkin lymphoma	4	1.67	2.40 [0.60, 6.27]
Non-Hodgkin lymphoma	19	1.09	17.45 [10.36, 27.50]
Central nervous system	4	3.71	1.08 [0.27, 2.82]
*After censoring post-transplant patients*			
All malignancies	15	16.72	0.90 [0.49, 1.49]
Leukaemia	7	3.19	2.19 [0.85, 4.59]
Lymphoma	0	2.59	–
Hodgkin lymphoma	0	1.56	–
Non-Hodgkin lymphoma	0	1.03	–
Central nervous system	3	3.54	0.85 [0.15, 2.54]

*IQR* interquartile range

In addition to the 41 malignancies, there were a further 33 diseases classified as benign or borderline malignancies, including nine lymphoproliferative disorders and nine intraepithelial neoplasia of the cervix/exocervix. In total, 73 patients developed a neoplasm, malignant or otherwise. The majority of the 25 tumours with clearly defined locations were in the abdomen/pelvis (n = 18) or head/neck (n = 6). There were no definite thoracic tumours (the location of one melanoma was listed as ‘trunk’).

## Transplantation

Among the cohort, 509 individuals received a transplanted organ before February 2014. The majority of these transplantations involved the heart only (80%), or heart and lungs (5%). The mean age at transplantation was 9.2 years. Twenty six malignancies developed among this patient group (63% of malignancies in the cohort) including 23 lymphomas (all cases of this disease). The mean age at diagnosis was 16.9 years for post-transplant malignancies, compared to 15.1 among non-transplant patients. The potential impact of transplantation on SIR was investigated by censoring observations for transplant recipients at the date of transplantation. This resulted in a reduction in the SIR to 0.90 for all malignancies combined (95% CI 0.49, 1.49) (Table 3). The SIR for leukaemia was essentially unchanged, however (2.19, 95% CI 0.85, 4.59). Thirteen patients developed benign or borderline malignancies, post-transplant, including 9 with lymphoproliferative disorder (100% of cases in the cohort). Three patients developed NMSC. None of the patients developing cervical intraepithelial neoplasia were identified as transplant recipients.

## Radiation exposure

Cohort members underwent a total of 17,154 recorded cardiac catheterizations and 4372 CT scans, up to February 2012 (i.e. 2 years before end of follow-up). The majority of patients had a single recorded catheterization (Table 1). Estimated organ doses are shown in Table 4. The mean ABM dose was 8.4 mGy. There were 384 patients with estimated cumulative bone marrow doses less than 0.1 mGy and 60 with doses over 100 mGy. The most common catheterizations were patent ductus arteriosus occlusion, coronary angiography, atrial septal defect occlusion and electrophysiology studies. The majority of CT scans in these patients were of the head, chest, or chest in combination with other body parts. A positive correlation was seen between the number of cardiac catheterizations and the number of CT scans (Spearman’s ρ = 0.252, 95% CI 0.233, 0.272). Transplant recipients underwent a total of 2395 recorded catheterization procedures and 1416 CT scans. The majority of these procedures (82%) and the majority of the associated dose (75%) (Table 4) occurred post-transplant, consistent with monitoring for rejection and coronary allograft vasculopathy.

**Table 4:** Estimated cumulative organ doses, up to 1st February 2012 (2 years prior to end of follow-up), from cardiac catheterizations for cohort members

	Breasts^a^	Lungs	Oesophagus	Thyroid	ABM	ABM (CC + CT)
*Mean organ dose (mGy) [IQR]*						
Whole cohort	33.1 [63.8]	42.9 [78.8]	26.2 [47.2]	1.6 [3.3]	6.6 [12.8]	8.8 [16.5]
Transplant recipients	49.4 [74.8]	80.7 [109.1]	48.3 [64.9]	2.3 [3.5]	15.8 [22.3]	28.3 [32.4]
Pre-transplant	16.5 [56.7]	20.0 [62.4]	11.5 [35.7]	0.7 [2.2]	3.1 [9.3]	6.6 [14.8]
Post-transplant	32.9 [53.6]	60.7 [94.4]	36.8 [56.8]	1.6 [2.8]	12.6 [20.6]	21.7 [29.2]
Cases^b,c^	63.7 [81.9]	80.6 [86.0]	43.3 [42.1]	2.0 [1.7]	14.2 [16.3]	20.9 [22.2]
Transplant recipient cases^b,c^	63.7 [81.9]	81.7 [78.7]	43.7 [38.9]	1.9 [1.7]	14.1 [13.3]	22.2 [20.8]
*Median organ dose (mGy) [IQR]*						
Whole cohort	14.1 [5.1, 33.5]	20.2 [8.2, 46.2]	13.0 [5.4, 27.2]	0.7 [0.3, 1.6]	3.1 [1.2, 6.6]	3.1 [1.3, 9.3]
Transplant recipients	19.0 [5.7, 68.5]	39.5 [14.5, 100.2]	25.6 [9.6, 60.7]	1.2 [0.4, 2.7]	7.7 [2.7, 19]	16.9 [6.4, 37.2]
Pre-transplant	0.0 [0.0, 7.0]	0.0 [0.0, 10.4]	0.0 [0.0, 5.7]	0.0 [0.0, 0.4]	0.0 [0.0, 1.7]	0.0 [0.0, 6.4]
Post-transplant	9.3 [2.1, 37.5]	23.3 [5.7, 72.1]	15.9 [3.4, 44.4]	0.7 [0.2, 1.8]	4.7 [1.0, 14.5]	11.1 [2.5, 28.7]
Cases^b,c^	34.0 [7.3, 85.5]	59.5 [22.5, 99.1]	35.1 [15.9, 58.3]	1.5 [0.7, 3.1]	10.2 [3.0, 19.2]	12.7 [3.8, 30.6]
Transplant recipient cases^b,c^	34.0 [7.3, 85.5]	69.8 [24.6, 102.9]	36.9 [16.1, 58.4]	1.4 [0.8, 2.8]	11.9 [4.3, 19.5]	14.6 [7.2, 31.8]

*ABM* active bone marrow, *ABM* (*CC* + *CT*) cumulative doses from both cardiac catheterizations and computed tomography combined, *IQR* interquartile range

^a^Female patients only

^b^Cumulative dose up to 2 years prior to diagnosis

^c^lymphohaematopoietic neoplasia, malignant and borderline, used in ERR models

The ‘post-latency’ SIR for malignancies developing at least 5 years following the first recorded cardiac catheterization (2 years for leukaemia or lymphoma), remained raised for all malignancies (3.01, 95% CI 2.09, 4.19) and lymphoma (9.15, 95% CI 5.66, 13.97) (Table 5) though with somewhat wider confidence intervals. After censoring observations for transplant recipients, the post-latency SIR for all malignancies was reduced to 0.98 (95% CI 0.48, 1.77). A sensitivity analysis was performed by calculating post-latency SIR after excluding all patients with cumulative ABM doses less than 0.1 mGy. This resulted in a slight increase in SIR for all malignancies to 3.04 (95% CI 2.11, 42.4) for the whole cohort, and 0.99 (95% CI 0.48, 1.79) after censoring transplant recipients.

**Table 5:** Standardized incidence ratio (SIR) for malignancies developing at least 5 years following the first recorded cardiac catheterization (2 years for leukaemia and lymphoma)

Disease	Observed	Expected3	SIR [IQR]
*All patients*			
All malignancies	36	11.98	3.01 [2.09, 4.19]
Leukaemia	4	2.31	1.73 [0.43, 4.53]
Lymphoma	22	2.40	9.15 [5.66, 13.97]
Hodgkin lymphoma	4	1.48	2.70 [0.68, 7.07]
Non-Hodgkin lymphoma	18	0.92	19.49 [11.39, 31.10]
Central nervous system	3	1.92	1.57 [0.28, 4.70]
*After censoring post-transplant patients*			
All malignancies	11	11.26	0.98 [0.48, 1.77]
Leukaemia	4	2.22	1.80 [0.45, 4.71]
Lymphoma	0	2.25	–
Hodgkin lymphoma	0	1.38	–
Non-Hodgkin lymphoma	0	0.87	–
Central nervous system	2	1.82	1.10 [0.09, 4.11]

*IQR* interquartile range

The ERR was calculated based on 36 malignant and borderline malignant lymphohaematopoietic neoplasia (29 among transplant recipients) and a total of 74,405 person-years (3446 for transplant recipients). Ignoring the transplant status interaction term, i.e. *β*_2_ = 0, the ERR was $\widehat{{\beta_{1} }} = 0.018$ mGy^−1^ (95% profile likelihood (PL) CI − 0.002, 0.096). The hat symbol over the beta parameter denotes the maximum likelihood estimator. Including the transplant status interaction term $\widehat{{\beta_{1} }} = 0.042$ mGy^−1^ and $\widehat{{\beta_{2} }} = -\, 0.021$. When transplant status information was not taken into account at all, i.e. where $\alpha_{7} = 0$ and *β*_2_ = 0, the ERR was $\widehat{{\beta_{1} }} = 0.542$ (95% PL lower bound: 0.104, Wald based upper bound: 1.807). Where this latter model was modified by censoring transplant patients post-transplant, the ERR was 0.149 mGy^−1^ (95% PL CI 0.001, 0.564). Omitting the absorbed dose information, i.e. *β*_1_ = 0 and $\beta_{2} = 0$, the risk effect of transplant status estimate was $\widehat{{\alpha_{7} }} = 4.370$, indicating that the risk of haematological neoplasia was almost 80 times larger for patients who underwent transplantation.

## Discussion

This is the largest study to date investigating cancer incidence among young people who have undergone cardiac catheterizations, and the first to include detailed radiation dose estimation and transplant registry linkage. The most interesting finding was the large apparent impact of transplantation on cancer rates in this patient group. Transplant recipients are treated with immunosuppressive therapies, including cyclosporine and tacrolimus [18], and receive relatively high radiation doses, especially post-transplant. Immunosuppression [14] and ionising radiation [19] are both well-known risk factors for cancer development and cannot be disentangled by SIR analysis alone. Our dose response analysis modelled the impact of transplantation in a similar manner to sex in similar radiation epidemiology studies, albeit with transplantation status as a time-dependent variable. The results of this analysis suggest radiation exposure alone does not account for the high rates of lymphohaematopoietic tumours among post-transplant patients, which in turn suggests immunosuppression is the dominant underlying cause. Furthermore, the apparent impact of transplantation was greatest for lymphoma and lymphoproliferative disorders; conditions strongly associated with immunosuppression [18, 20, 21], though relatively weakly associated with radiation exposure (e.g. [19]). In contrast, rates for leukaemia, a disease strongly associated with radiation exposure [19, 22] but less so with immunosuppression [20], were almost unaffected by censoring transplant recipients.

The absence of any cancers of the lungs, breasts and oesophagus was expected, despite the high mean dose to these tissues (Table 4). These diseases are rare below age 35 years [23], even among individuals exposed to elevated radiation levels [24]. Only 135 cohort members had reached the age of 35 years by February 1st 2014. The lack of cancers of the breasts, lungs or oesophagus should not be regarded as evidence of no risk, at this stage of follow-up. The drive to keep radiation exposures as low as reasonably achievable must, therefore, be maintained.

We were only able to gather information on cardiac catheterizations and CT. What may be termed ‘dark dose’; exposure from other sources such as general radiography and nuclear medicine, could be considerable for certain individual patients, despite contributing only a small proportion of the total cumulative dose for the whole cohort. Uncertainties in dose estimates include measurement error (i.e. kerma area product) and uncertainty in the conversion factor from which organ doses are derived, due to variation in beam angle, field size and x-ray energy. A further source of uncertainty relates to the lack of dose indicator for 21% of examinations, meaning organ doses needed to be estimated based on era-specific median doses for which dose indicators were available. For bone marrow, this figure was 3.1 mGy for examinations prior to 2003. Doses vary considerably from one procedure to the next, however. For example, the interquartile range for ABM dose was 1.65–5.60 mSv, while the 95th percentile was 18.3 mGy. Future work will attempt to quantify these uncertainties using 2D Monte Carlo techniques, allowing them to be incorporated into risk estimates.

Other than transplantation, we had limited information on other potential tumour-predisposing syndromes, such as neurofibromatosis or ataxia telangiectasia. Around 55% of examination records included a notes field in which relevant history could be recorded, while other details could be obtained from the cause of death for patients who died. Four malignancies (all leukaemia) were found among patients identified as having Down syndrome, versus 0.14 expected. Very few cases were identified among children with the most serious congenital heart defects, including hypoplastic left/right ventricles (1 case) transposition of the great arteries or tetralogy of Fallot (no malignancies, one NMSC). This analysis is limited, however, by the small proportion of the cohort for whom examination notes were available. Future studies would benefit from linkage with congenital anomalies registries.

## Comparison with previous studies

Three other studies have investigated cancer incidence or mortality among children undergoing cardiac catheterizations. A retrospective cohort study of 4861 children who underwent cardiac catheterisations between 1946 and 1968 in Ontario, Canada, reported 5 cancer deaths were observed, compared to 4.8 expected, after 13 years of follow-up [1]. A further study [2] using the same cohort (reduced to 3915 members due to exclusion of patients living outside the study area) reported a standardised mortality ratio (SMR) of 1.2 (90% CI 0.6, 2.3), based on 7 cancer deaths versus 5.7 expected. The SIR was 0.75 (0.3, 1.2), based on 13 cancer cases observed versus 17.3 expected. As with the current study, a number of cancers were reported in sites remote from the heart including the tongue, testis (two cases), prostate, ovary, cervix, colon and brain. Modan et al. [3] reviewed details of 674 Israeli children who underwent cardiac catheterizations between 1950 and 1970. Dose records were unavailable for 90% of cohort members. The SIR was 2.3 (95% CI 1.2, 4.1), based on 11 cases compared to 4.75 expected, including 4 lymphomas and 3 melanomas. At least six of the tumours occurred in locations remote from the heart (testis, prostate, bladder, inguinal lymph nodes and melanomas of the groin and lower limb). The location of the others was unclear. Interestingly, all cancers occurred in males, who represented 56% of the cohort.

Other studies have focused on cancer rates among people with CHD, irrespective of whether or not they underwent cardiac catheterizations. A recent study [5] focussed on 31,961 Taiwanese patients of all ages, identified from insurance records, diagnosed with CHD between 1998 and 2006. The SIR for all cancer sites was 1.45 (95% CI 1.25, 1.67). Around half of these patients had undergone a catheterization procedure (48.9%) while 18.9% underwent CT scanning. The most common cancers were haematological (SIR = 4.04). Lymphomas were not analysed separately. Heart transplants are carried out in Taiwan [25], though it is unclear how many patients had undergone this procedure. Other studies have reported increased rates of cancer among individuals with cardiovascular malformations, as part of wider studies investigating other forms of congenital diseases [4, 6, 7]. Although the results of our analysis are broadly consistent with previous studies, it should be noted that the current cohort was established from hospital records of patients undergoing cardiac catheterizations, rather than from registers of congenital malformations.

Our SIR figures can also be compared to more general studies of children exposed to diagnostic x-rays (i.e. not specifically cardiac patients). Hammer et al. [26] reported SIRs of 0.99 for all cancers (95% CI 0.79, 1.22), 1.08 for leukaemia (95% CI 0.74, 1.52) and 0.97 for lymphoma (95% CI 0.52, 1.66) for 92,957 German children postnatally exposed to diagnostic x-rays. Average estimated doses were very low (mean effective dose = 0.137 mSv, compared to 13.1 mSv in the current study). The SIR for all cancers was similar to that of the current study after censoring transplant patients. Our SIR for leukaemia was higher, though statistically compatible with that of Hammer et al. [26]. Focussing only on CT scans, Krille et al. [27, 28] reported raised SIRs for all cancers (1.82, 95% CI 1.29, 2.50), lymphoma (2.96, 95% CI 1.42, 5.45) and leukaemia (1.72, 95% CI 0.89, 3.01). After excluding patients at increased risk of cancer, SIR figures were reduced to 1.54 for all cancers (95%: CI 1.05, 2.19), 1.79 for leukaemia (95% CI 0.92, 3.12) and 1.85 for lymphoma (95% CI 0.68, 4.02). The impact of this change on lymphoma SIR was much smaller than found in the current study, although Krille et al. [27, 28] did not have the benefit of transplant registry linkage. Mean cumulative bone marrow doses (11.7 mGy) were a little higher than in the current study (8.8 mGy).

The average equivalent dose to bone marrow from natural sources, including gamma rays and radon has been estimated to be 1.5 mSv per year at age 1 year, falling to 1.3 mSv per year at age 15 years [29]. Thus, the mean cumulative ABM dose from recorded medical exposures in this group (8.3 mGy, equivalent to 8.3 mSv) is comparable to around 5–6 years of background radiation. Background gamma exposure has been associated with a relative risk (RR) of 1.08 mGy^−1^ (95% CI 1.02, 1.16) for leukaemia and non-Hodgkin lymphoma [22]; a figure higher than, but statistically compatible with the ERR for lymphohaematopoietic neoplasia in the current study.

## Conclusion

Cancer rates among children and young adults who undergo x-ray guided cardiac catheterizations are high, compared to the general population. Despite transplant recipients making up less than 5% of the cohort, these individuals contributed more than 60% of observed malignancies. While immunosuppression may be the most likely explanation for this, the higher radiation doses received by transplant recipients, post-transplant, may also be a contributing factor. Further analysis is planned with an enlarged cohort, congenital anomaly registry linkage, extended follow-up and pooling with cohorts from other countries (e.g. [30]). We recommend that future studies of the cancer risks from cardiac x-ray procedures take steps to identify transplant recipients, ideally through registry linkage.
